# MicroRNA-derived Fragment Length Polymorphism Assay

**DOI:** 10.1038/srep09356

**Published:** 2015-03-20

**Authors:** Xiaoping Xie, Fang Tang, Zhao Yang, Yaoyi Zhang, Zihao Feng, Yu Yang, Xiujin Wu, Feifei Zhang, Jie Zhu, Kai Xu

**Affiliations:** 1 Dujiangyan Medical Center, Chengdu, Sichuan, 611830, China; 2 Chengdu Nuoen Biotechnologies, Inc., Chengdu, Sichuan, 610041, China; 3 Chengdu University of Traditional Chinese Medicine, Chengdu, Sichuan, 610075, China

## Abstract

MicroRNA (miRNA) studies are experiencing a transition from basic research applications to clinical applications. However, the lack of reliable and sensitive miRNA detection methods has become a bottleneck in the process. Here, we report an absolute quantification method based on the competitive PCR amplification of specific miRNAs and synthetic RNA spike-ins in a single reaction. RNA spike-ins are quantified as dynamic RNA copy number standards and are used to measure selected miRNAs free from the effects of intra-assay variables, including those from individual sample sources. Combined with the size differentiation power of capillary electrophoresis, the content of miRNAs was reproducibly measured, with verifiable detection limits of 10–46 copies over 5-log detection ranges. The direct measurements of miRNAs from 168 human serum samples and their considerable value as a diagnostic for bronchopneumonia and bronchiolitis demonstrate the potential of the assay in clinical applications.

miRNAs regulate cell proliferation and differentiation^1^. The expression profiles of pathogen activated miRNAs reveal the onset of pathogenic events or the outcome of disease^2^,^3^,^4^,^5^,^6^. Surprisingly, miRNAs were also readily detected in a wide range of body fluids, specifically as stable protein-associated and microvesicle-associated miRNAs in blood^7^,^8^,^9^. Although little is currently known about how miRNAs are released into blood, there is enormous interest in using circulating miRNA signatures as minimally invasive biomarkers for diagnostic and prognostic applications. For example, hsa-miR-122-5p is a highly liver specific miRNA that has 52,567 copies enriched in each liver cell, but the serum baseline levels of this miRNA are very low in healthy individuals^10^,^11^. When liver tissues are damaged, the serum hsa-miR-122-5p levels could be detected several hundred-fold higher than the baseline a few hours after the event^12^,^13^,^14^,^15^. These features make it a potential biomarker for liver injuries with unmatched sensitivity and specificity compared with the regulatory agency endorsed ALT test^16^. In general, miRNA signatures can potentially serve as minimally invasive biomarkers in the diagnosis and prognosis of many diseases, once clinically suitable methods and standard operation procedures (SOP) for miRNA measurements are established.

There are over 2,000 human miRNA species registered in miRbase release 21^17^. Mature miRNAs are non-coding, single-stranded RNA molecules, 22 nucleotides (nt) in length, with significant diversity in nucleotide composition and complex secondary structures that pose a challenge to their purification and detection. Relative miRNA abundance in biological samples can be measured by a number of commercially available assay kits based on different miRNA detection platforms, such as reverse transcriptase quantitative polymerase chain reaction (RT-qPCR), hybridization-based microarray and next generation sequencing (NGS)^11^,^18^,^19^.

Recent analyses have revealed that the performance of these methods varies, with each platform having strengths and weaknesses in solving practical detection problems^19^,^20^,^21^,^22^,^23^. RT-qPCR methods are the most sensitive detection technologies available and are the *de facto* method of choice for the quantification of circulating miRNAs, due to the low abundance of miRNA in blood. However, that status has been challenged by studies revealing the use of RT-qPCR resulted in high inter-assay imprecision with serum samples^20^,^21^. Further studies found that a few pre-analytical and post-analytical variables were accountable for the inter-assay variances. Unfortunately, these variables, such as RNA-extraction methods, RNA quantification protocols, threshold Cq reporting method and data normalization, are essential elements of the RT-qPCR technology.

Acknowledging the inefficiency of RNA-extraction methods and the sample differences that introduced analytic variances in current detection methodologies, we have developed the **miR**NA-derived **F**ragment **L**ength **P**olymorphism (miRFLP) assay for the simultaneous quantification of multiple miRNAs and synthetic RNA spike-ins in a single reaction. The methodology improves detection reliability by eliminating intra-assay variables, including those from individual sample sources. Using this method, we were able to reproducibly determine target miRNA copy numbers in as little as 0.2 μl of serum with improved inter-assay precision.

## Results

### miRFLP assay design

**Figure 1:**
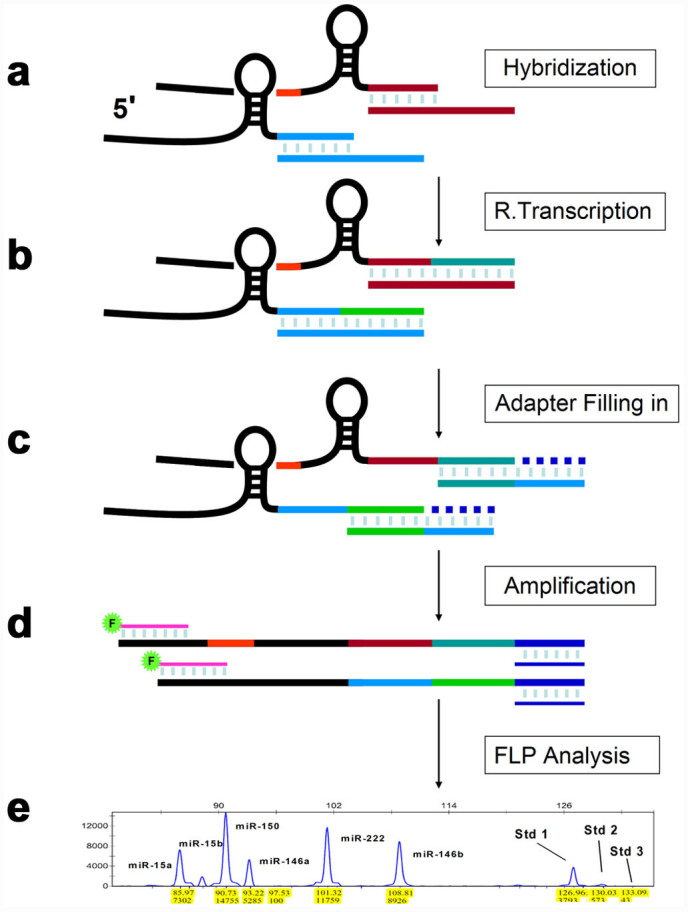
Overview of miRFLP assay. (a) The mixture of miRNAs and iRCSs is subjected to hybridization in solution with sequence-specific omega primers. (b) RT is performed. (c) After RNA removal, the newly extended portions of matched omega primers are used as probes in hybridization with template adapters. The hybrids are extended by DNA polymerase using adapter sequences as templates. (d) Competitive PCR amplification of correctly assembled fragments. (e) DNA fragment length analysis is performed by automated capillary electrophoresis.

To offset the effect of intra-assay variables, we developed a strategy using synthetic RNAs at different concentrations as intrinsic RNA copy standards (iRCSs). When the quantification of selected miRNAs and iRCSs is performed competitively in a single reaction, the measurements of miRNA and iRCSs should be closely correlated to their input copy numbers, free from the influence of most intra-assay variables. To achieve this goal, we used structured omega primers (Supplementary Fig. 1) for size-coded, multiplexed small RNA reverse transcription. With size-coded omega primers, selected miRNAs and iRCSs are converted to cDNA fragments in different lengths simultaneously. The work flow of miRFLP assay is comprised of five steps, as illustrated in Figure 1. The assembling of cDNAs relies on the correct base pairing of omega primers with both miRNAs and adapters in sequential order. The properly structured DNA fragments are flanked by a pair of universal PCR target sites; thus, a pair of PCR primers can be used for the competitive amplification of all target fragments in the same reaction.

RT-qPCR assays measure target amplicons either by probe hybridization or fluorescence staining, where specific amplicons are confirmed by annealing temperatures in later method. In cells, there are several transcripts for a given miRNA in mature, precursor and primary transcript forms with variable lengths. Many miRNAs are also composed family members that differ by only a few nt in non-seed regions. The sequence similarity of miRNA transcripts poses significant challenges to probe hybridization specificity and temperature annealing analyses. In the miRFLP assay, the targets are recognized by amplicon lengths. The lengths of assembled cDNAs are pre-defined by the combined lengths of the omega primers, adapters and gaps (or minus overlapped nt) formed by the RNA templates through RT and adapter filling-in reactions. Omega primers with 11-nt probes are used for the RT of miRNAs, and the newly synthesized oligonucleotides are again used as probes for oligonucleotide extension on template adapters. The use of two shorter probes provides higher thermodynamic gaps between matched and mismatched targets in each reaction steps. Furthermore, because the reverse transcribed oligonucleotides are copies of miRNA templates, template adapters could be specifically designed for different transcripts. Non-specific DNA fragments in different lengths can be distinguished by capillary electrophoresis, with a length variance as little as 0.2 nt. We examined two DNA fragments, 123.05 and 125.17 nt in length, in 40 PCR reactions; the fragments were acquired using an ABI 3730xl DNA analyzer with standard deviations of 0.05 and 0.04 nt, respectively. The superior reproducibility and resolution of capillary electrophoresis provide a powerful length differentiation method with multiplexed capacity for the identification of target DNA fragments. With this powerful signal identification resolution, the hybridization of omega primers and miRNA targets can be conditioned in favor of sensitivity rather than specificity. In addition, dozens of miRNAs can be clearly multiplexed as fragments in sizes between 80 to 140 nt, at 3 nt apart.

The cDNA assembly during RT and the adapter filling-in steps are performed quantitatively with linear relationships, but the conversion efficiencies vary upon the probe compositions and miRNA secondary structures. DNA templates with small length variances (approximately 100 bp) were equally amplified by competitive PCR^24^. In miRFLP assays, DNA template sizes are usually limited to 140 bp, with a length variance from 3–60 bp, due to synthesis errors of long oligonucleotides. The competitive amplifications, coupled with optimized PCR conditions, have normalized conditions for the equal amplification of DNA templates derived from miRNAs and iRCSs alike in a single test. The absolute measurements of a target miRNA in different tests are affected by intra-assay variables, such as sample impurities and sample sources, but their abundance over dynamic iRCS scales are constants of the miRNA input copy numbers when standard amounts of iRCSs are used. Thus, the relative abundance of the target miRNA in different samples can be directly compared, free from the effects of sample-bound variables. The method used to calculate the miRNA relative abundance is explained in detail in the online Methods.

### Assay sensitivity and specificity

**Figure 2:**
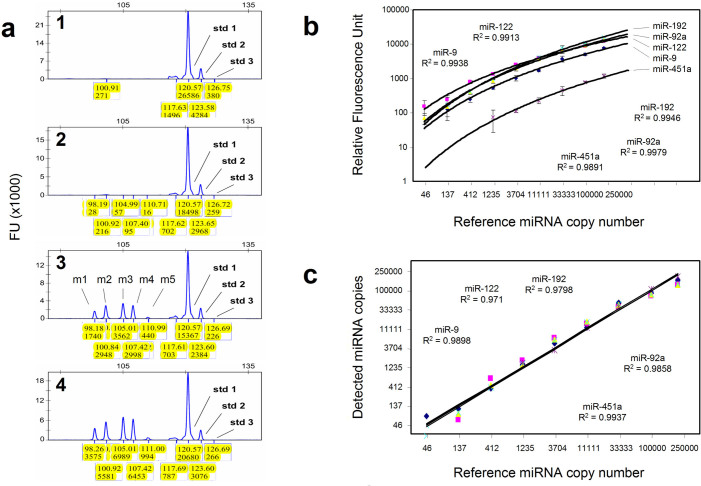
Quantitative detection of miRNA UR by miRFLP assay. (a) Plot view of fluorescence peaks in panel 1, 2, 3 and 4 represented the measurements of 0, 46, 11111 and 33333 copies of UR containing equimolar of m1: miR-9-5p, m2: miR-122-5p, m3: miR-192-5p, m4: miR-92a-3p and m5: miR-451a. The std 1, std 2 and std 3 were annotated for peaks derived from iRCSs, which formed a RNA copy scale for each test. The peaks of 117.6 nt were designated for RNA spike-ins. The numbers in yellow boxes were lengths (upper, nt) and fluorescence unit (lower, FU) of DNA fragments. (b) The detected miRNA relative fluorescence units (RFU) were plotted against their corresponding reference miRNA copy numbers used. UR miRNA in amounts of 46 to 250000 copies were tested as standard curves. The standard curves for miRNA copy number conversion were fit with Power regression. (c) The dynamic detection ranges with regression coefficient estimates for assay accuracy are shown for each miRNA. The data used to construct these graphs are listed in Supplementary Tables 2 and 3.

The circulating levels of hsa-miR-9-5p, hsa-miR-122-5p, hsa-miR-192-5p, hsa-miR-92a-3p and hsa-miR-451a were studied as biomarkers for drug induced liver injuries (DILI)^16^. The miRFLP DILI Assay was designed for the detection of the above miRNAs, plus an RNA spike-in and 3 iRCSs, simultaneously (Supplementary Table 1). The measurements were made with 3-fold serially diluted miRXplore Universal Reference (UR) in the range of 46–250,000 copies in triplicate, and the chromatogram plots for measurements of selected reactions are presented in Figure 2a. The miRNA relative abundances were plotted against the synthetic miRNA input copy numbers as standard curves in Figure 2b. Power regressions were predicted as best fits by SPSS statistics with coefficients of >0.99 for each miRNA, except hsa-miR-451a. The serum hsa-miR-451a levels varied in wide ranges, depending on sample hemolysis status^20^. Thus, lower primer concentrations were used to validate the unpredictable nature of the present circulating hsa-miR-451a. At miRNA input levels of 412 copies and above, the averaged intra-assay coefficients of variations (CVs) were 21.78%, 16.16%, 14.42% and 13.46% for hsa-miR-9-5p, hsa-miR-122-5p, hsa-miR-192-5p and hsa-miR-92a-3p, respectively. When 137 copies of synthetic RNAs or less were tested, the averaged intra-assay CVs were inflated to 57.85%, 86.62%, 51.49% and 66.04%, respectively, due to assay sensitivity and possibly widened operational variances (Supplementary Table 2). For those reasons, miRNA relative abundances of less than 400 input copies were used for assay sensitivity verification, not to calculate standard curves.

To determine assay accuracy, the miRNA relative abundances were tested on 3-fold serially diluted URs and converted to miRNA copy numbers using the standard curves plotted in Figure 2b. The detected miRNA copy numbers were plotted on a log scale against the expected synthetic miRNA inputs with linear regression coefficients of >0.97 for each miRNA (Fig. 2c). The assay accuracy CVs followed the same trend as that of intra-assay variances (Supplementary Table 3). The miRFLP assays demonstrated a detection range of 5 orders of magnitude with sensitivities between 10 and 46 copies of target miRNAs.

The miRFLP assay specificity is ensured by three layers of discriminating mechanisms. First, the specific conversion of miRNAs to cDNAs through hybridization between omega primers and miRNA templates. Second, the successfully extended omega primers from the RT step are selectively hybridized with matching adapter oligonucleotides at the fill-in step. Third, the superior resolution of capillary electrophoresis guarantees the detection of the correct fragments. To demonstrate this specificity, individual members of hsa-let-7 family were tested with miRFLP hsa-Let-7 assay (Supplementary Table 4). The results are listed in Supplementary Table 5, with the target miRNAs detected set as 100%. The low levels of non-specific detection were observed across all hsa-let-7 members. The sequences of hsa-let-7 family members were listed in Supplementary Table 6 for comparison.

### miRFLP assay on purified RNA samples

Total RNA was extracted from cultured cells with TRIzol reagent following the vendor's instructions. RNA concentrations were determined using a Qubit 2.0 fluorimeter (Life Technologies), and RNA integrity numbers (RIN) were measured as 9.40, 9.60 and 9.30 for RNA from A549, HeLa and H1299 cells (ATCC) using an Agilent Bioanalyzer 2100. RNA samples, including 10-fold serially diluted A549 RNA, were tested in triplicate with the miRFLP miR-92ab assay (Supplementary Table 7). Table 1 lists the machine readout (fluorescence unit), the converted relative fluorescence unit (RFU) and the calculated miRNA concentration of each test. The power equations of 0.1343X^1.1384^ and 2.4234X^1.3068^, fit from standard curves of serially diluted URs, were used to calculate the miRNA copy numbers of hsa-miR-92a and hsa-miR-92b from their respective RFUs. The miRNA concentrations measured from serially diluted A549 RNA samples demonstrated the dynamic detection range of the miRFLP assay with CVs of 12.6% and 10.1% for hsa-miR-92a and hsa-miR-92b, respectively. However, inflated variations were also observed when fewer miRNA targets were tested in diluted A549 RNA. This phenomenon was consistent with assay accuracy variations reported in the previous section. Thus, technical repeats would help improve the assay accuracy when miRNA in low abundance is tested.

### Serum-direct miRFLP assay

Because RNA extractions affected miRNA quantification dramatically^20^,^21^,^25^, a reliable serum-direct detection method would have a significant impact on assay accuracy and reproducibility and would save both time and costs. There are a few reports of direct miRNA detection on sera or cell lysates by RT-qPCR^26^,^27^, and the inter-sample variation was the major hurdle for their broader use. Using iRCSs as references, the miRFLP assay was developed to reduce intra-assay variances introduced by different samples. Serum miRNAs were readily detected by the miRFLP IM assay (Supplementary Table 8) applied to heat denatured serum directly. When sera were treated at 95°C, the detected miRNA levels peaked between 30 seconds to 1 minute of incubation and decreased gradually with incubation time (Supplementary Table 9). Most miRNAs were below detection limits after 10 minutes of incubation. Different temperatures were tested for optimal miRNA measurements, and the serum denaturation conditions of 75°C for 5 minutes was chosen for its balanced miRNA release and the narrower assay variances observed (Supplementary Table 10). Four serum samples were measured with serum-direct miRFLP IM assay, and the results were comparable to those obtained from purified RNAs of the same serum (Supplementary Fig. 2).

**Figure 3:**
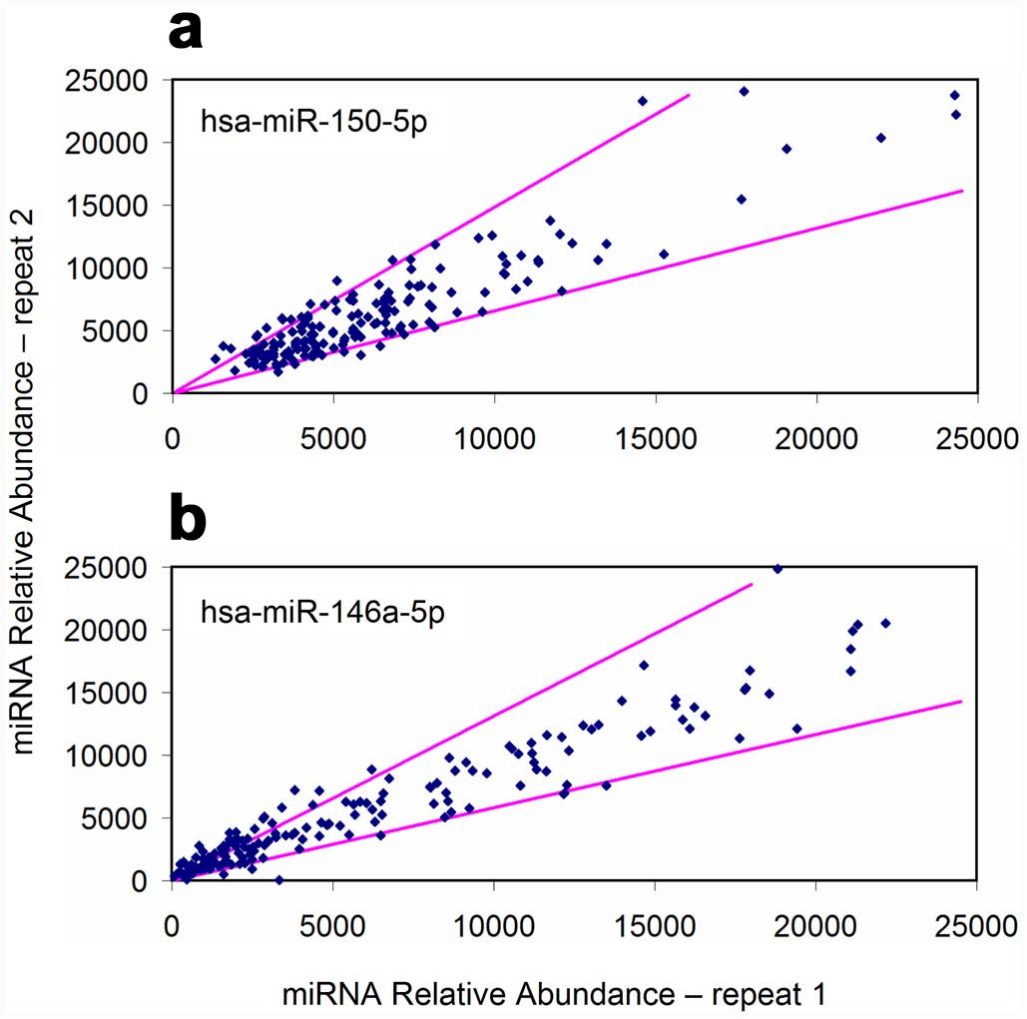
Reproducibility of serum-direct miRFLP assay. miRNA relative abundances from two independent tests on 168 serum samples were plotted against each other for (a) hsa-miR-150-5p and (b) hsa-miR-146a-5p. The pink lines indicated 50% deviation boundaries from the mean values. The percentages of data points within 50% deviation boundaries (between pink lines) were 90.5%, 84.5% and 80.4% for hsa-miR-150-5p, hsa-miR-146a-5p and hsa-miR-222-3p, respectively. The percentages of data points within 100% deviation boundaries were 66.1% and 77.8% for hsa-miR-146b-5p and miR-181a-5p (data not shown).

Heat incubation might result in serum clotting from sample to sample. To test the adaptability of optimized serum lysis condition, 168 serum samples were diluted 1:20 in serum lysis buffer and incubated at 75°C for 5 minutes. No serum clotting was observed at the end of tests. The miRNA measurement results for two independent repeats were scatter plotted against each other to show run-to-run assay reproducibility. The representative graphs of hsa-miR-150 and hsa-miR-146a are displayed in Figure 3. The results indicated that the majority of repeated measurements for hsa-miR-150, hsa-miR-146a and hsa-miR-222 were within 50% of deviation from their averages and that reproducibility compared favorably with that of well-executed RT-qPCR tests, where the inter-assay imprecision on serum samples was reported to be 1.43-fold to 3.66-fold^20^,^21^. The repeat results of hsa-miR-181a and hsa-miR-146b possessed larger variations due to their low expression levels in tested sera. It seems reasonable to predict that assay variations would be narrowed when the expression levels of those miRNAs are elevated.

### Serum miRNA levels of asthma patients

**Figure 4:**
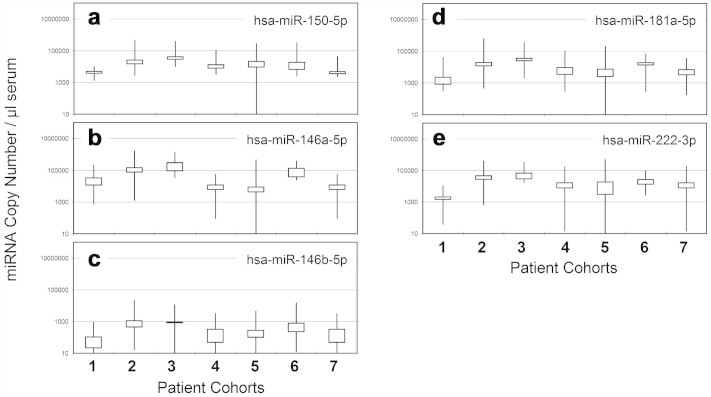
Serum miRNA concentrations in healthy and patient cohorts. The *x*-axis corresponds to patient cohorts: 1. Healthy (n = 13), 2. Bronchopneumonia (n = 41), 3. Bronchiolitis (n = 17), 4. Asthma (n = 32), 5. Asthmatic Bronchiolitis (n = 27), 6. Acute Asthmatic Bronchiolitis (n = 8), and 7. Others (n = 24). The boxes represented 75% of data points for each cohort. The vertical line represents the high-low values of each cohort. miRNA copies in 1 μl of serum were reported for (a) hsa-miR-150-5p; (b) hsa-miR-146a-5p; (c) hsa-miR-146b-5p; (d) hsa-miR-181a-5p and (e) hsa-miR-222-3p.

miRNAs are associated with innate immune response (hsa-miR-146a, hsa-miR-146b and hsa-miR-150) and asthma (hsa-miR-222 and hsa-miR181a)^3^,^5^,^28^,^29^. As a pilot study for identifying asthma miRNA biomarkers, the serum-direct miRFLP IM assay was used to measure those miRNAs in sera collected from 13 healthy individuals, 125 pediatric patients diagnosed with asthma or bronchopneumonia and 24 patients with mild coughs or non-asthmatic food allergies. Our results in Figure 4 confirm the stable presence of innate immune response and asthma miRNAs in serum samples and elevated serum levels in clinically categorized cohorts. The independent sample *t*-test analyses determined that hsa-miR-146a was the best indicator to differentiate all cohorts (p < 0.01), except for cohorts of bronchopneumonia vs. bronchiolitis and acute asthmatic bronchiolitis vs. healthy. Hsa-miR-150 and hsa-miR-222 distinguished the combined cohorts of bronchopneumonia and bronchiolitis vs. all others (p < 0.01). Hsa-miR-146b showed significant difference in distinguishing asthma cohort from the others. Hsa-miR-181a provided marginal differentiation power for bronchopneumonia and bronchiolitis from other cohorts (p < 0.05). The diagnostic performance of each miRNA was further analyzed with receiver operating characteristic (ROC) curves (Supplementary Fig. 3, 4 and 5). The area under ROC curves (AUC) was estimated at 0.877 (95% CI, 0.82–0.934) for hsa-miR-146a in diagnosing combined cohorts of bronchiolitis and bronchopneumonia from others. However, the most clinically meaningful diagnostic between cohorts of asthmatic bronchiolitis and asthma was not identified in the miRNAs tested in this pilot study.

### miRNA signature in DILI rats by CCl4

**Figure 5:**
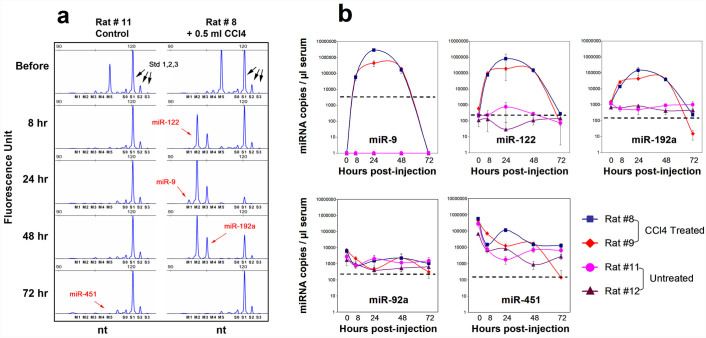
Alteration of rat serum miRNAs induced by CCl4 administration. (a) Chromatogram plots of miRNA measurements of sera collected from 2 rats over the indicated time courses. m1: rno-miR-9a-5p; m2: rno-miR-122; m3: rno-miR-192-5p; m4: rno-miR-92a-3p; m5: rno-miR-451-5p. S0: RNA spike-in; S1, S2 and S3: iRCSs. (b) Time-dependent profiling of serum miRNA levels from 2 rats with a single CCl4 injection. Dashed lines indicate the detection limits for respective miRNAs in 1 μl of serum. Tissue-enriched and RBC-enriched miRNAs (miR-9: brain, cervix. miR-122: liver. miR-192: small intestine, kidney, liver. miR-92a: generic including RBC. miR-451: RBC).

To probe the changes of serum miRNA expression in animal models with DILI, 10 SD rats were injected intraperitoneally with a single dose of 0.5 ml of carbon tetrachloride (CCl4). Blood draws were performed before administration and then at 8, 24, 48 and 72 hours after CCl4 administration. We examined the miRNA concentrations in available sera using miRFLP DILI assay. The baselines of rno-miR-122, rno-miR-192-5p and rno-miR-92a-3p were low at 208 ± 205, 730 ± 258 and 1263 ± 757 copies per μl of serum (mean ± SD, n = 10), respectively, with variable serum rno-miR-451-5p concentrations and undetectable serum rno-miR-9a-5p. The serum levels of tissue-enriched rno-miR-9a-5p, rno-miR-122 and rno-miR-192-5p increased sharply 8 hours after injection, reached peaks of 2954553 ± 177171, 804887 ± 38485 and 146009 ± 5424 copies per μl of serum at 24 hours (n = 7), and then dropped to baseline levels at 72 hours (n = 2). The miRNA measurements by miRFLP DILI assay are demonstrated in chromatogram plots of miRNA measurements over the time course for rat #8 and control rat #11 in Figure 5a. Two-tailed Pearson correlations analyses were performed on the same miRNA measurements (Supplementary Table 11). Rno-miR-92a-3p and rno-miR-451-5p had highly correlated expression patterns with coefficients of 0.911, and this correlation implied their red blood cell (RBC) origin, in agreement with previous reports in human plasma samples (R = 0.77)^30^. The alterations of serum rno-miR-9a-5p, rno-miR-122 and rno-miR-192-5p over the time course were significantly synchronized in response to the injuries induced by CCl4 (Fig. 5b and Supplementary Table 11). No cross-correlation was observed between tissue-enriched miRNAs and RBC-associated miRNAs. The substantial improvements in miRNA quantification led to a more subtle revelation of the underlining mechanisms with less experimental animal usage.

## Discussion

Inconsistent miRNA quantification results are often reported by different platforms and between research groups^12^. Most miRNA detection methods that are used in practice lack the sensitivity to reach sub-attomolar levels, except for RT-qPCR methods that report miRNA relative abundances as fold changes rather than absolute copy numbers. These fold changes must be converted to target copy numbers by standard curves, which are performed on serially diluted synthetic RNAs separately. Because several issues should be considered to obtain quality RT-qPCR results, the Minimum Information for Publication of Quantitative Real-Time PCR Experiments (MIQE) guidelines were suggested to ensure transparent and comparable qPCR results^31^. The same issues surrounding RT-qPCR are exaggerated further by the short lengths of miRNAs, which make it more difficult to enforce MIQE guidelines in miRNA detection involving RT-qPCR technology.

A recent review of 154 circulating miRNA signatures for 26 different tumor types from publications during last 5.5 years demonstrate the technological pitfalls and limitations that contributed to the reproducibility issues in circulating miRNA biomarker research^32^. Ironically, since the first reports of stable miRNAs in circulating body fluids in 2008, many of the pre-analytical and analytical variables that affect circulating miRNA analyses have been identified in depth, such as sample sources, RNA-extraction, inter-assay imprecision and data normalization^20^,^21^,^22^,^25^. Fundamentally, there are potential biases in the fold change reporting method employed by RT-qPCR when considering low-abundance miRNAs^33^. In such cases, the reliable measurements of miRNA absolute copy numbers could provide better assessments of pathological phenomena. In previous work, the serum hsa-miR-122-5p levels were correlated to liver fibrosis stages^13^. Although the sera of chronic hepatitis C (CHC) carriers with normal ALT levels showed elevated hsa-miR-122-5p, the miRNA levels of CHC carriers displayed no difference from those levels observed in acute liver failures^34^. Here, the miRFLP assay of our acute DILI models revealed that serum rno-miR-122-5p levels were up regulated by CCl4 and went down drastically within 72 hours. With a shorter half-life (12–24 hours), serum miRNAs can be further explored to differentiate acute and chronic status.

The core value of the miRFLP assay is the simultaneous measurement of iRCS and spike-ins with target miRNAs. This strategy appears to be easily adaptable to the multiplexable digital counting methods of NGS technologies. The deep sequencing reads of small RNA libraries constructed with coded oligonucleotides as quantitative calibrators has been demonstrated successfully in the discovery of placental-specific miRNA in both maternal and newborn circulations, and their relative abundance have been determined using approximately 500 μl of serum or plasma^35^. However, substantial improvements must be made to the NGS platform for it to become a prime tool for miRNA quantification. For one, the detection limits of NGS platforms are well over 10,000 miRNA copies even with PCR amplification^19^,^22^. In addition, a long list of questions must be addressed, such as the sequence-dependent bias introduced by miRNA structures, the long processes of small RNA library construction and the requirement of pure RNA as starting material. Nevertheless, deep sequencing technologies hold great promise for future miRNA quantification.

The robust miRFLP assay was developed for absolute quantification of miRNAs by combining the sensitivity of competitive RT-PCR with the high-fidelity length differentiation of capillary electrophoresis analysis. Furthermore, the presence of a co-amplified iRCSs sets the assay apart from traditional RT-qPCR methods and makes it strategically possible to detect absolute miRNA copy numbers directly in biological samples, such as serum, free from sample variances. The miRNA references for each assay can be standardized to facilitate objective inter-laboratory data interpretation and to fulfill the regulatory-required multicenter data validation prior to any diagnostic applications. The serum-direct miRFLP assay reduces serum usage to sub-microliter levels and takes advantages of the stability of miRNA in serum, which can be stored at −20°C for 2–4 years^36^ and withstand multiple freeze-thaw cycles. The demonstrated initial tests on 168 individual serum samples paves the way for the further evaluation of serum-direct miRFLP assay in broad clinical applications.

## Methods

### Blood collection and serum preparation

The study was approved by the Institutional Review Board of the Dujiangyan Medical Center. Written, informed consent was obtained from every individual or legal custodian before samples were collected. The SOP for Collection of Serum Blood samples from the Early Detection Research Network (EDRN) was followed for blood collection and serum preparation with minor modification. Serum supernatants were separated by additional centrifugation of 13,000 × *g* for 10 minutes at 4°C to remove possible cellular debris, as demonstrated by McDonald^20^. The supernatants were transferred into new tubes in small aliquots and kept in liquid nitrogen for long-term storage or −20°C for <20 months^36^.

### Animal models

Animal experiments were conducted at the Laboratory Animal Center, Sichuan University. Animal maintenance and treatment conformed fully to the National Institutes of Health Guide for Animal Welfare of China. The study was approved by the Institutional Animal Care and Use Committee of Sichuan University, Sichuan, China. According to World Health Organization - Environmental Health Criteria 208, the LD50 values of CCl4 ranged from 1.77 ml–63.4 ml/kg for rats. To generate acute liver injury models, ten 8-week-old SD rats were injected with 0.5 ml of CCl4 intraperitoneally without fasting. Two rats were injected with 0.5 ml PBS as controls. Eight rats failed to reach the 72-hour end point, and 2 animals survived. Then, 50–200 μl of blood were repeatedly collected at the designated time points before and after drug treatments. Rats were euthanized after the last blood collection, and liver necrosis were confirmed visually in CCl4-treated rats.

### RNA isolation

TRIzol LS reagent (Life Technologies) was prepared in 300 μl aliquots containing 12 million copies of RNA spike-ins and 100 ng of bacterial RNA as carriers. The TRIzol aliquots were frozen at −20°C until use. There was a previous report that quantitative recovery of plasma RNA would not extend over high input volume^25^, and we found that the observation also held true for serum samples. With 300 μl of TRIzol, the highest human serum volumes for quantitative RNA recovery fell between 20 μl to 50 μl, varying upon individuals, and the highest rat serum volumes were at 5 μl to 10 μl (data not shown). In this study, 5 μl of rat serum or 10 μl of human serum was diluted into each aliquot. The RNA isolation procedure was performed according to the vendor's protocol, except the RNA precipitations were stored overnight at −20°C before centrifugation at 13,000 × *g* for 30 minutes at 4°C. Purified RNAs were dissolved in 10 μl of RNA Storage Buffer containing 10 ng of bacterial RNA and stored at −20°C. Then, 2 μl of RNA (equivalent of 2 μl of serum) was tested in each reaction. Because bacteria lack true miRNA^37^, we used bacterial RNA purified from DH5a as carrier RNA for miRFLP assays to avoid possible miRNA contaminants from miRNA-bound, biologically generated RNA carriers, such as glycogen, yeast RNA or MS2 phage RNA^25^,^38^. The below detectable signals in miRFLP assay were consistently observed with all tested miRNAs using bacterial RNA carrier in concentration up to 100 ng/ul as diluents.

### Oligonucleotide design and preparation

A sample omega primer with a stem-loop is illustrated and explained in Supplementary Figure 1. The secondary structures of omega primers were confirmed using the RNA folding program on the mfold Web Server^39^. The adapter serves as a template for the extended omega primers and provides the addition of a universal PCR target site. The probe sequence is usually designed as 11 nt long and is identical to the 5′ sequence of a target miRNA.

Sense PCR primer sequence: [6 Fam]- GTGCTGAGTCACGAGGTATTCTA

Antisense PCR primer sequence: CACCGACAGGAGACCTGTTCT or CACGGAGGTGTTATCCGAAGA oligonucleotides of PAGE-purified grade were purchased from GenScript, Nanjing, China. Omega primers were dissolved at a concentration of 5 μM in 1x TE (pH 8.0) and then denatured at 95°C for 1 minute before slowly cooling to room temperature at 1°C per minute. Adapters were prepared at a concentration of 5 μM in 1x TE, and 50 μM PCR primer solutions were prepared using 1x TE. All primers were stored at −20°C in the dark.

miRXplore Universal Reference v1.0 was purchased from Miltenyi Biotec Inc, Germany. UR contains equimolar mixture of more than 950 synthetic miRNAs, including all miRNAs used in this study.

### RNA synthesis

iRCSs, RNA spike-in and individual hsa-let-7 oligoribonucleotides were purchased from GenScript, Nanjing, China in PAGE-purified grade.

iRCS #1: ACCGUACAUCU ugu UGCAUAUCCGA

iRCS #2: ACCGUACAUCU ugaagu UGCAUAUCCGA

iRCS #3: ACCGUACAUCU ugaaucagu UGCAUAUCCGA

iRCS #4: ACCGUACAUCU UCAUAAUCCGA

iRCS #5: ACCGUACAUCU UGCAUAUCCGA

iRCS #6: ACCGUACAUCU UAGUAAUCCGA

RNA spike-in: ACCGUACAUCU UGCAUAUCCGA

The sequence information of each synthetic hsa-let-7 miRNA is listed in Supplementary Table 6.

### miRFLP assay

First, 2 μl of RNA samples was mixed with 4 μl of serum lysis buffer containing 2.5x MMLV RT buffer (Takara), 0.25% of Tween-20 (Sigma-Aldrich), 3 × 10^6^ copies of iRCSs and 0.1 μl of RNase Inhibitor (RI, New England Biolabs). Next, 2 μl of probe mix containing 10 nM of omega probe for each target miRNA was added to the mixture. Hybridization was performed on a PCR block with a 10-minute pretreatment at 55°C, which was then subjected to 5 cycles of 1 minute incubation at 55°C and 5 minutes at 30°C. The reactions were continued with additional 5 cycles of incubation, with 1 minute at 30°C and 5 minutes at 8°C. The reactions were held at 4°C for 20 minutes. Then, 2 μl of enzyme mix containing 0.5 μl of MMLV reverse transcriptase (Takara) and 50 nM of dNTP (Sigma-Aldrich) were added to each hybridization reaction. RT was performed at 20°C for 20 minutes and then at 37°C for 10 minutes. The reactions were stopped with 5 minutes of incubation at 85°C and held at 4°C.

Subsequently, 30 μl of cDNA extension buffer containing 10 nM of adapters, 0.1 μg of RNase A (Sigma-Aldrich), 0.5 μl of JumpStart Taq DNA polymerase and 15 μl of 2x JumpStart PCR buffer (Sigma-Aldrich) was pipetted into each 10 μl of RT reaction. The cDNA extension reactions were performed with a 2-minute denaturation at 95°C, followed by 10 minutes of RNase digestion at 60°C, another 10 cycles of 5 minutes of incubation at 55°C and 1 minute at 40°C, and a final incubation of 5 minutes at 72°C.

Then, 5 μl of cDNA extension reaction was mixed with 25 μl of PCR solution containing 15 μl of 2x PCR buffer, and 0.3 μl of sense and antisense PCR primer mix. PCR was performed with a 2-minute activation at 95°C followed by 40 cycles of 15 seconds at 95°C and 3 minutes at 68°C, 10 minutes at 72°C and 1 hour of incubation at 60°C. PCR products were diluted 1:20 in 1x TE and analyzed using an ABI 3730xl DNA analyzer at Chengdu Genegle Biotechnologies.

### Serum-direct miRFLP assay

Serum samples were diluted 1:20 in serum lysis buffer, and the mixtures were incubated at 75°C for 5 minutes. After cooling to 4°C, 2 μl of iRCSs (3 × 10^6^ copies), RNA spike-in and 0.1 μl of RI were added to 4 μl of serum lysates. Then, 2 μl of probe mix containing 10 nM of omega probe for each target miRNA was added to each serum RNA mixture. The reactions were forwarded to the hybridization step as described in the miRFLP Assay section.

### miRNA relative abundance

The measurement of serially diluted PCR products by the ABI 3730xl DNA analyzers confirmed that quadratic and power regression represented the best fits between fluorescent measurements and dilution factors (Supplementary Table 12). Power regressions were fit to iRCS input copies, and their FUs were detected in each test. The miRNA relative fluorescence units were converted from detected miRNA FU using the power equations acquired in the same test.

### Statistical analysis

The independent sample *t*-test, ROC curves and AUCs, Pearson correlation tests and the estimation of best regression fits were carried out using IBM SPSS (Statistical Package for the Social Sciences) Statistics v20. The standard deviation and power regression models were performed in Microsoft Excel 2002. The coefficient of variation was calculated as standard deviation/average.

## Author Contributions

All authors confirmed they have contributed to the intellectual content of this paper with final approval of the publication. X.X. and K.X. conceived the concept and prepared the manuscript. X.X., F.T., Z.Y. and K.X. designed the experiments and analyzed data. F.T., Y.Z., Z.F., Y.Y. and K.X. developed the miRFLP assay. F.T., Y.Z., Z.F., Y.Y., X.W., J.Z. and F.Z. provided experimental ideas and technical support. X.X. and Z.Y. collected clinical samples and performed analyses on clinical data.

## Supplementary Material

Supplementary Information Supplementary Information

## Figures and Tables

**Table 1:** The measurement of hsa-miR-92a and hsa-miR-92b from purified RNA samples

	Fluorescence Unit					RFU		miRNA copy number/ng RNA			
RNA sample, amount	iRSC #4	iRSC #5	iRSC #6	miR-92b	miR-92a	miR-92b	miR-92a	miR-92b	C.V	miR-92a	C.V
A549 RNA, 3.12 ng	27418	682	112	960	13251	5635	151696	**7,110**	12.61%	**394,996**	6.35%
	31659	690	128	1246	15984	6934	166186				
	32118	662	133	1103	15867	5929	164125				
A549 RNA, 0.312 ng	32073	689	168	370	4029	1273	27763	**8,681**	35.34%	**345,449**	25.50%
	27276	405	143	159	2313	668	19071				
	30185	496	119	212	3316	963	27564				
A549 RNA, 0.0312 ng	31859	928	206	68	1149	93	4306	**8,248**	24.83%	**307,042**	86.27%
	31378	1880	241	146	372	145	553				
	31741	1087	186	87	1507	131	6093				
Hela RNA, 0.222 ng	31356	1297	264	1487	7360	4567	46128	**54,097**	27.03%	**747,141**	40.34%
	7536	198	39	184	946	3058	25880				
	31718	804	162	776	4509	3144	30931				
H1299 RNA, 0.250 ng	31569	892	141	300	3488	963	22175	**7,195**	56.09%	**509,963**	24.15%
	31825	1209	272	221	5702	295	32215				
	31733	1004	199	322	4895	752	30252				
