# Supplementary material for: MicroRNA-derived Fragment Length Polymorphism Assay
Source: Sci Rep. 2015 Mar 20;5:9356. doi: 10.1038/srep09356 (PMC4366852; doi:10.1038/srep09356)
Supplement: Supplementary Information [file srep09356-s1.pdf]

Supplementary Information for:

microRNA-derived Fragment Length Polymorphism Assay

Xiaoping Xie[1†], Fang Tang[2†], Zhao Yang[1], Yaoyi Zhang[2], Zihao Feng[2], Yu Yang[2], Xiujin Wu[2], Feifei Zhang[2], Jie Zhu[3] and Kai Xu[2,*]

Contents of Supplementary Figures and Tables:

Supplementary Figure 1 The schematic representation of omega primer.

Supplementary Figure 2 Comparison of miRNA measurements in purified RNA and in serum samples.

Supplementary Figure 3 The ROC curves of selected miRNAs for diagnosis of bronchopneumonia, bronchiolitis and acute asthmatic bronchiolitis patients.

Supplementary Figure 4 The ROC curves of miRNAs for diagnosis of asthma patients.

Supplementary Figure 5 The ROC curves of miRNAs for diagnosis of asthma and asthmatic bronchiolitis patients.

Supplementary Table 1 Oligonucleotides used in miRFLP DILI assay.

Supplementary Table 2 Intra-assay variance of miRFLP DILI assay.

Supplementary Table 3 Intra-assay accuracy of miRFLP DILI assay.

Supplementary Table 4 Oligonucleotides used in miRFLP hsa-let-7 assay.

Supplementary Table 5 Specificity evaluation of miRFLP assay on hsa-let-7 family members.

Supplementary Table 6 hsa-let-7 miRNA sequence comparison chart.

Supplementary Table 7 Oligonucleotides used in miRFLP miR-92ab assay.

Supplementary Table 8 Oligonucleotides used in miRFLP IM assay.

Supplementary Table 9 Incubation duration affects the quantification of serum miRNAs.

Supplementary Table 10 Optimization of serum-direct miRFLP assay.

Supplementary Table 11 Correlation coefficients of tissue-enriched miRNAs and RBC-associated miRNAs in sera of rats over the time course of CCl4 treatment.

Supplementary Table 12 The responses of ABI 3730xl DNA analyzer detector.

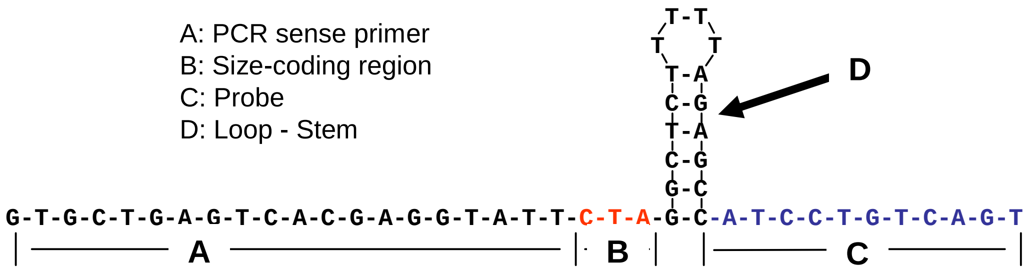

Supplementary Figure 1 The schematic representation of omega primer. A: PCR primer target site. B: size-coding region. The numbers of nucleotides in this region can be edited to change the length of final PCR products. C: Probe region. The nucleotide sequence used here is reverse complimentary to the 3´ end of target RNA. D: stem-loop region. The nts fold into a strong stem loop at low temperature to separate probe sequence from other functional regions structurally. The partitioning of functional groups allows independent alteration of nts in probe and size-coding regions. Properly formed omega primers also demonstrate additional features that benefit the priming of short RNAs, such as target location preference on RNA 3´ ends, reduced primer dimerization and minimum internal initiation etc (Patent No.: PCT/CN2013/070525).

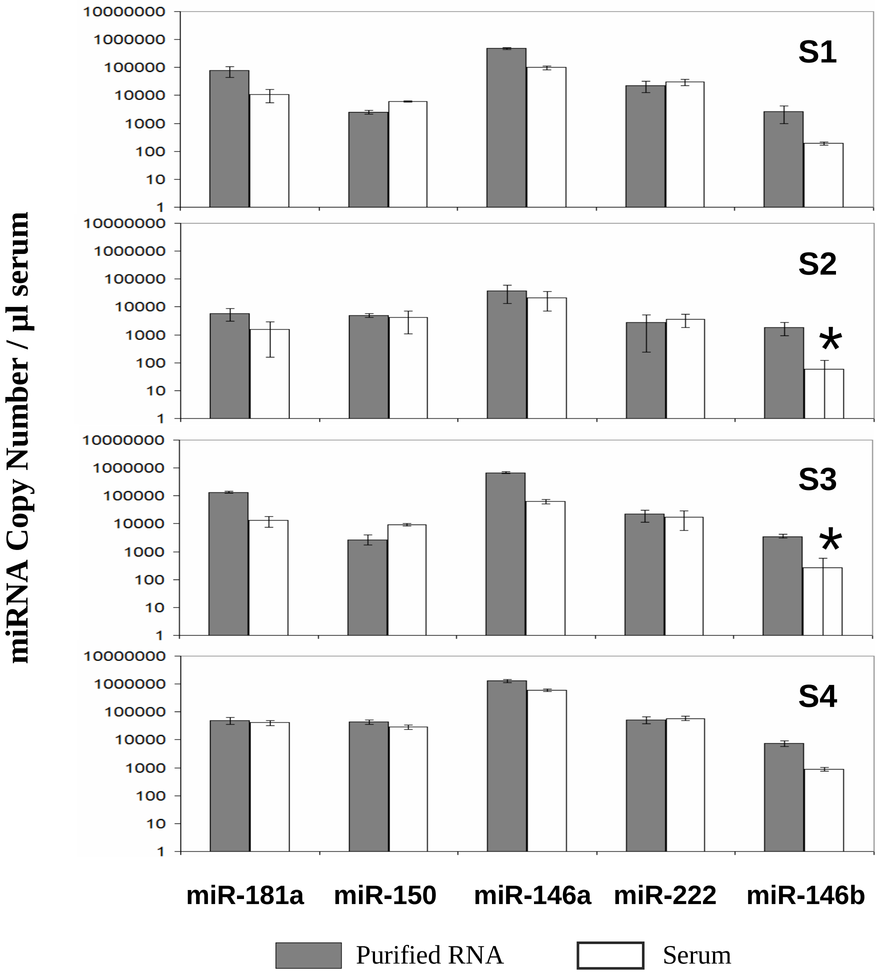

Supplementary Figure 2 Comparison of miRNA measurements in purified RNA and in serum samples. The miRNA concentrations obtained from purified RNA and serum samples displayed significant correlations by 2-tailed Pearson correlation analyses. The correlations for four tested serum samples were: 0.969 $p < 0.01$ (S1); 0.954 $p < 0.05$ (S2); 0.962 $p < 0.01$ (S3) and 0.978 $p < 0.01$ (S4), respectively.

*: measurements were close to detection limit.

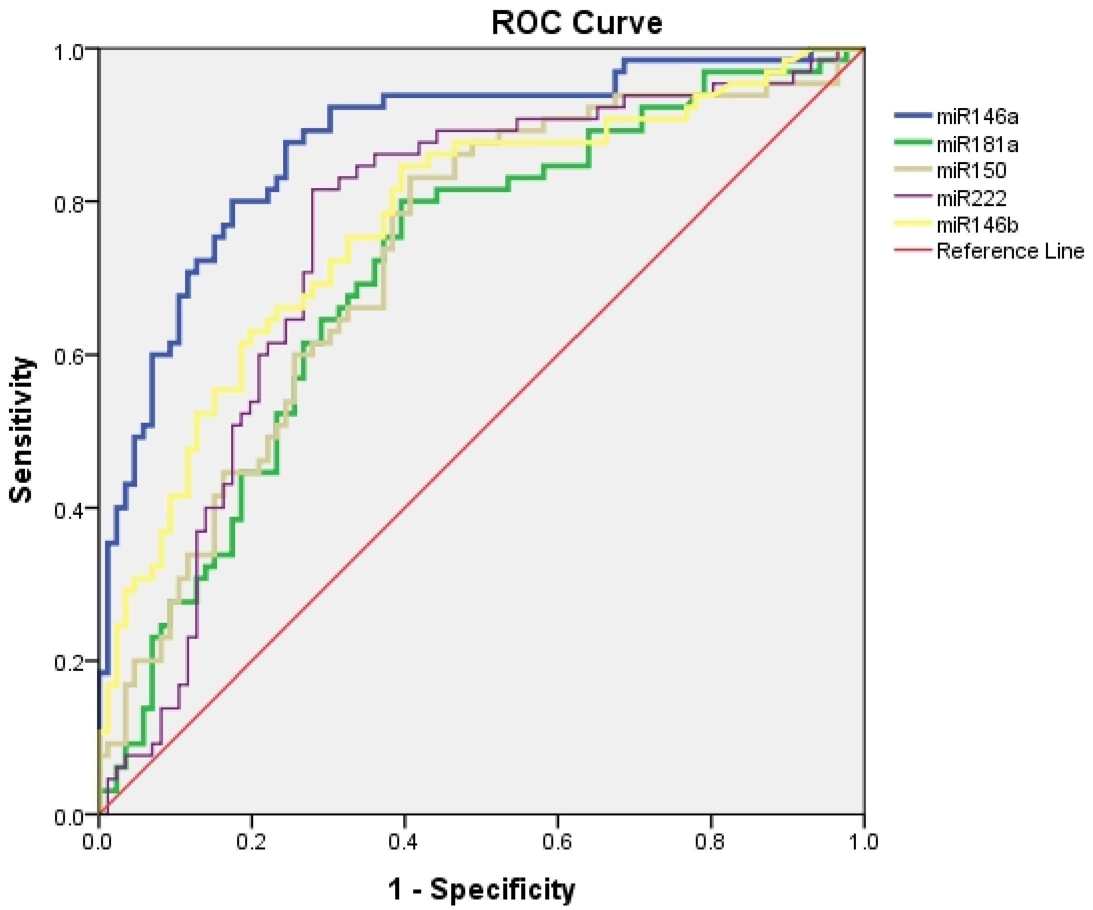

Supplementary Figure 3 The ROC curves of selected miRNAs for diagnosis of bronchopneumonia, bronchiolitis and acute asthmatic bronchiolitis patients. The ROC curves of hsa-miR-181a, hsa-miR-150, hsa-miR-146a, hsa-miR-222 and hsa-miR-146b on their predictive values to differentiate the cohorts of bronchopneumonia, bronchiolitis and acute asthmatic bronchiolitis from other cases were drawn in 1-Specificity plot. The AUC values of tested miRNAs were: hsa-miR-146a (0.877, $p < 0.001$, 0.820 – 0.934), hsa-miR-181a (0.709, $p < 0.001$, 0.626 – 0.793), hsa-miR-150 (0.720, $p < 0.001$, 0.646 – 0.809), hsa-miR-222 (0.753, $p < 0.001$, 0.673 – 0.834) and hsa-miR-146b (0.772, $p < 0.001$, 0.695 – 0.848).

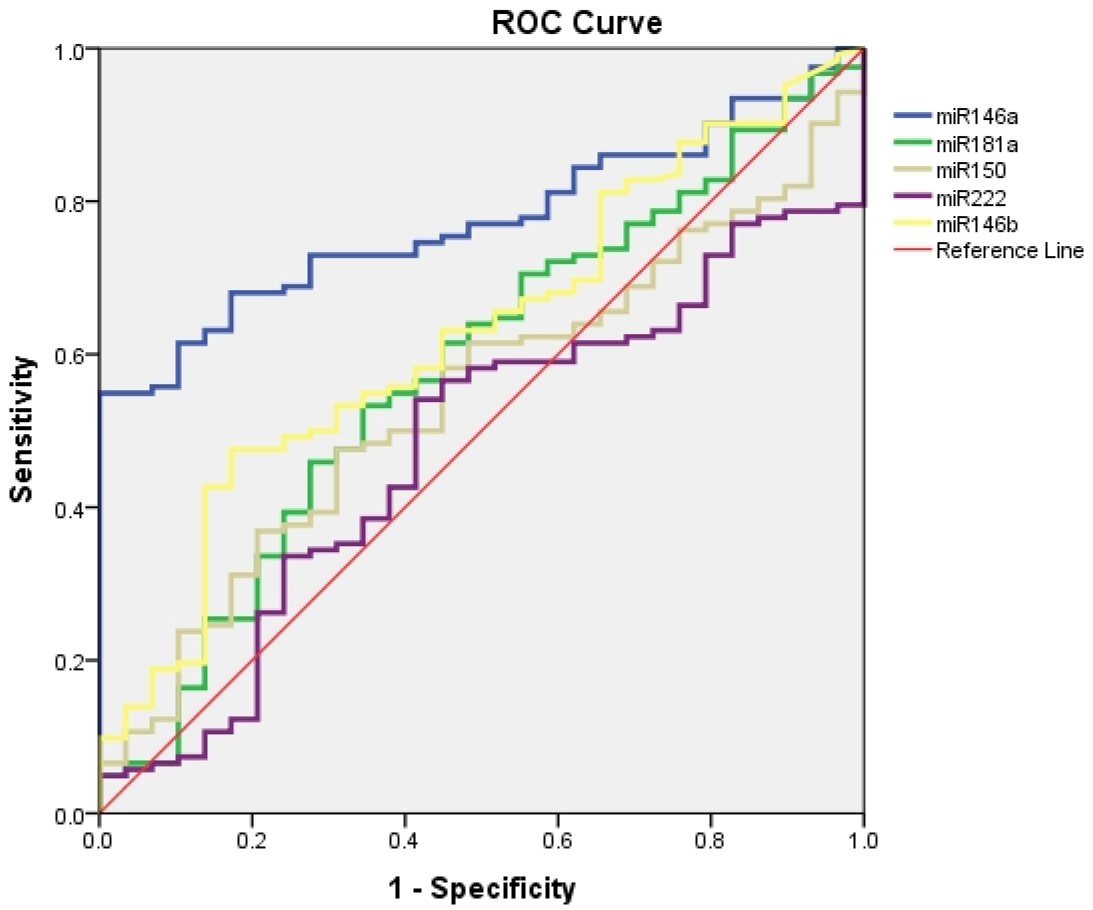

Supplementary Figure 4 The ROC curves of miRNAs for diagnosis of asthma patients. The ROC curves for hsa-miR-181a, hsa-miR-150, hsa-miR-146a, hsa-miR-222 and hsa-miR-146b on their predictive values to differentiate asthma patients from other cases were drawn in 1-Specificity plot. The AUC values for tested miRNAs were: hsa-miR-146a (0.775, $p < 0.001$, 0.701 – 0.848), hsa-miR-181a (0.580, $p = 0.181$, 0.466 – 0.694), hsa-miR-150 (0.543, $p = 0.476$, 0.438 – 0.648), hsa-miR-222 (0.477, $p = 0.702$, 0.369 – 0.585) and hsa-miR-146b (0.626, $p = 0.035$, 0.52 – 0.733).

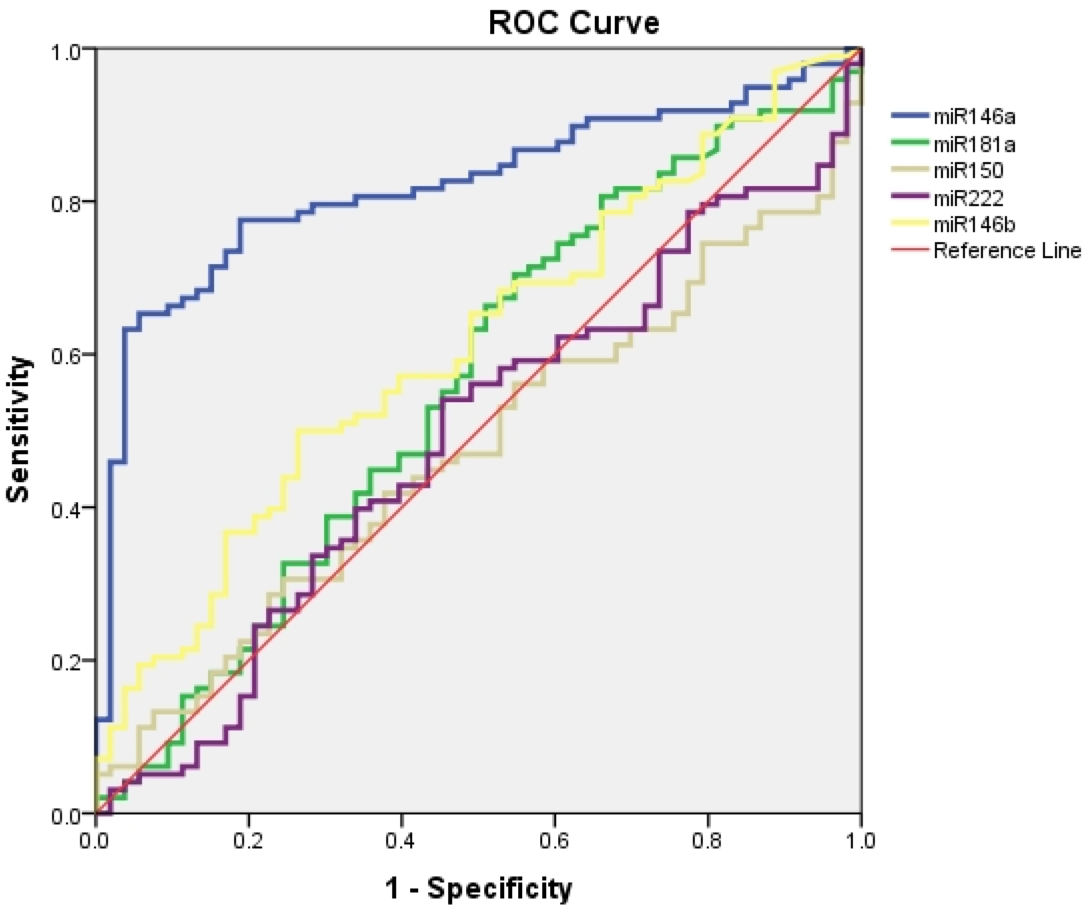

Supplementary Figure 5 The ROC curves of miRNAs for diagnosis of asthma and asthmatic bronchiolitis patients. The ROC curves for hsa-miR-181a, hsa-miR-150, hsa-miR-146a, hsa-miR-222 and hsa-miR-146b on their predictive values to differentiate asthma and asthmatic bronchiolitis patients from other cases were drawn in 1-Specificity plot. The AUC values for tested miRNAs were: hsa-miR-146a (0.820, $p < 0.001$, 0.753 – 0.888), hsa-miR-181a (0.559, $p = 0.232$, 0.461 – 0.657), hsa-miR-150 (0.473, $p = 0.591$, 0.381 – 0.566), hsa-miR-222 (0.487, $p = 0.800$, 0.392 – 0.583) and hsa-miR-146b (0.611, $p = 0.047$, 0.519 – 0.703).

Supplementary Table 1 Oligonucleotides used in miRFLP DILI assay. The sequences of omega primers, the targeted miRNA or iRSC molecules, the sequences of 3´ adapters and the expected amplicon sizes are listed below.

RNA Name	Omega Primer	miRNA sequence	RNA Copy per Rx	3' Adapter	Fragment Length
	5' – 3'	5' – 3'		5' – 3'	expected
**iRCS #1**	GTGCTGAGTCACGAGGTATTCTA TGAATACCTTCAACTTGCAGTTACTGCAAG TCAT GGCACGCTTCTTAGCGTGCC TCGGATATGCAA	ACCGUACAUCU ugu UGCAUAUCCGA	3 X 10^6	CACCGACAGGAGACCTGTTCT ACCGTACATCT	124.27nt
**iRCS #2**		ACCGUACAUCU ugaagu UGCAUAUCCGA	5 X 10^5		127.36nt
**iRCS #3**		ACCGUACAUCU ugaaucagu UGCAUAUCCGA	8.3 X 10^4		130.30nt
**RNA spike-in**		ACCGUACAUCU UCAUAAUCCGA	variable		121.23nt
**hsa-miR-9-5p / rno-miR-9a-5p**	GTGCTGAGTCACGAGGTATTCTATTGTTCTTGCAGTT GGCACGCTTCTTAGCGTGCC TCATACAGCTAG	UCUUUGGUUAUCUAGCUGUAUGA	variable	CACCGACAGGAGACCTGTTCT TCTTTGGTTATCTA	101.10nt
**hsa-miR-122-5p / rno-miR-122**	GTGCTGAGTCACGAGGTATTCTATTGTTCTTGCAGTT AAGA GGCACGCTTCTTAGCGTGCC CAAACACCATTG	UGGAGUGUGACAAUGGUGUUUG	variable	CACCGACAGGAGACCTGTTCT TGGAGTGTGAC	103.78nt
**hsa-miR-192-5p / rno-miR-192-5p**	GTGCTGAGTCACGAGGTATTCTATTGTTCTTGCAGTT AAGATTAG GGCACGCTTCTTAGCGTGCC GGCTGTCAATT	CUGACCUAUGAAUUGACAGCC	variable	CACCGACAGGAGACCTGTTCT CTGACCTATGAA	107.41nt
**hsa-miR-92a-3p / rno-miR-92a-3p**	GTGCTGAGTCACGAGGTATTCTATGTTCTTGAGTTAT ATTCA GGCACGCTTTCATTAGCGTGCC G GAGGCCGGGA	UAUUGCACUUGUCCCGGCCUGU	variable	CACCGACAGGAGACCTGTTCT GAC TATTGCACTTG	110.53nt
**hsa-miR-451a / rno-miR-451-5p**	GTGCTGAGTCACGAGGTATTCTATTGTTCTTGCAGTT AAGATTAGTTACA GGCACGCTTCTTAGCGTGCC AACTCAGTAATG	AAACCGUUACCAUUACUGAGUU	variable	CACCGACAGGAGACCTGTTCT AAACCGTTACC	113.35nt

*: Average MW (308.95 Delton) of dG, dC, dA and dT is counted as 1 nt. MW of 5' Fam: 474.5.

Supplementary Table 2 Intra-assay variance of miRFLP DILI assay. 3-fold serially diluted UR were assayed independently in triplicates and the CVs for each miRNA were calculated from the triplicates at different reference miRNA input levels.

miRNA UR copy number	Intraassay coefficients of variations				
	miR-9	miR-122	miR-192	miR-92a	miR-451a
**250,000**	15.0%	6.4%	5.4%	1.4%	22.3%
**100,000**	13.9%	6.2%	3.3%	6.0%	11.8%
**33,333**	34.1%	33.8%	12.5%	22.5%	48.2%
**11,111**	16.8%	15.4%	11.0%	16.6%	32.8%
**3,704**	29.6%	13.8%	26.7%	17.0%	16.1%
**1,235**	13.5%	16.0%	21.5%	6.1%	94.5%
**412**	29.6%	21.7%	20.6%	24.6%	
**137**	71.6%	76.4%	44.6%	78.8%	173.2%
**46**	44.1%	96.8%	58.4%	53.2%	

Supplementary Table 3 Intra-assay accuracy of miRFLP DILI assay. 3-fold serially diluted UR were assayed independently in triplicates and the CVs between detected and expected miRNA copies were calculated at different reference miRNA input levels.

miRNA UR copy number	Variation Coefficients of detected vs. expected				
	miR-9	miR-122	miR-192	miR-92a	miR-451a
**250,000**	39.7%	34.9%	52.0%	50.9%	18.0%
**100,000**	26.8%	24.7%	32.5%	34.8%	2.6%
**33,333**	31.1%	13.1%	12.3%	16.4%	15.1%
**11,111**	2.5%	12.3%	24.4%	23.0%	1.4%
**3,704**	20.8%	32.7%	35.7%	32.1%	17.3%
**1,235**	8.7%	6.4%	3.3%	5.1%	38.2%
**412**	14.2%	12.2%	17.1%	21.1%	
**137**	12.8%	80.3%	52.1%	33.6%	62.9%
**46**	64.9%	76.0%	58.3%	13.6%	

Supplementary Table 4 Oligonucleotides used in miRFLP hsa-let-7 assay. The sequences of omega primers, the targeted miRNA or iRSC molecules, the sequences of 3´ adapters and the expected amplicon sizes are listed below.

RNA Name	Omega Primer	miRNA sequence	RNA Copy per Rx	3' Adapter	Fragment Length
	5' – 3'	5' – 3'		5' – 3'	expected
**iRCS #1**	GTGCTGAGTCACGAGGTATTCTA TGAATACCTTCAACTTGCAGTTACTGCAAG TCAT GGCACGCTTCTTAGCGTGCC TCGGATATGCAA	ACCGUACAUCU ugu UGCAUAUCCGA	3 X 10^6	CACCGACAGGAGACCTGTTCT ACCGTACATCT	124.20nt
**iRCS #2**		ACCGUACAUCU ugaagu UGCAUAUCCGA	5 X 10^5		127.10nt
**iRCS #3**		ACCGUACAUCU ugaaucagu UGCAUAUCCGA	8.3 X 10^4		130.17nt
**RNA spike-in**		ACCGUACAUCU UCAUAAUCCGA	variable		121.23nt
**hsa-miR-98**	GTGCTGAGTCACGAGGTATTCTA a GGCACGCTTCTTAGCGTGCC AACAATACAACT	UGAGGUAGUAA GUUGUAUUGUU	variable	CACCGACAGGAGACCTGTTCT AG TGAGGTAGTAAG	88.73nt
**hsa-let-7d**	GTGCTGAGTCACGAGGTATTCTA ACA GGCACGCTTCTTAGCGTGCC AACTATGCAACC	AGAGGUAGUAG GUUGCAUAGUU	variable	CACCGACAGGAGACCTGTTCT TCT AGAGGTAGTAGG	90.73nt
**hsa-let-7e**		UGAGGUAGGAG GUUGUAUAGUU	variable	CACCGACAGGAGACCTGTTCT ATGAGT TGAGGTAGGAGG	93.46nt
**hsa-let-7a**		UGAGGUAGUAG GUUGUAUAGUU	variable	CACCGACAGGAGACCTGTTCT ATAATACTC TGAGGTAGTAG DTT	96.78nt
**hsa-let-7b**	GTGCTGAGTCACGAGGTATTCTAACTTCTAA GGCACGCTTCTTAGCGTGCC AACCACACAAC	UGAGGUAGUAG GUUGUGUGGUU	variable		100.63nt
**hsa-let-7c**	GTGCTGAGTCACGAGGTATTCTAACTTCTAACAA GGCACGCTTCTTAGCGTGCC AACCATACAAC	UGAGGUAGUAG GUUGUAUGGUU	variable		102.70nt
**hsa-let-7f**	GTGCTGAGTCACGAGGTATTCTAACTTCTAACAATC AACA GGCACGCTTCTTAGCGTGCC AACTATACAAT	UGAGGUAGUAG AUUGUAUAGUU	variable		109.63nt
**hsa-let-7g**	GTGCTGAGTCACGAGGTATTCTAACTTCTAACAATC AACTTCA GGCACGCTTCTTAGCGTGCC AACTGTACAAA	UGAGGUAGUAG UUUGUACAGUU	variable		111.55nt
**hsa-let-7i**	GTGCTGAGTCACGAGGTATTCTAACTTCTAACAATC AACTACATAA GGCACGCTTCTTAGCGTGCC AACAGCACAAA	UGAGGUAGUAG UUUGUGCUGUU	variable		115.64nt

*: Average MW (308.95 Delton) of dG, dC, dA and dT is counted as 1 nt. MW of 5' Fam: 474.5.

Supplementary Table 5 Specificity evaluation of miRFLP assay on hsa-let-7 family members. Individual synthetic hsa-let-7 miRNAs were tested with miRFLP hsa-let-7 assay and the percentages of non-targeted copies over the targeted copies were shown to indicate non-specific crosstalks between hsa-let-7 family members. The targeted miRNA member was set as of 100% in each series.

		**The cross-detection of let-7 family members**								
		**hsa-miR-98**	**hsa-let-7a**	**hsa-let-7b**	**hsa-let-7c**	**hsa-let-7d**	**hsa-let-7e**	**hsa-let-7f**	**hsa-let-7g**	**hsa-let-7i**
**Synthetic let-7 miRNA**	**hsa-miR-98**	**100.0%**	0.0%	0.0%	0.0%	0.0%	0.1%	0.0%	0.0%	0.0%
	**hsa-let-7a**	0.0%	**100.0%**	0.2%	0.3%	0.0%	0.0%	0.7%	0.0%	0.0%
	**hsa-let-7b**	0.0%	4.2%	**100.0%**	12.4%	0.0%	0.0%	0.3%	0.1%	0.0%
	**hsa-let-7c**	0.0%	2.7%	0.4%	**100.0%**	0.0%	0.0%	1.6%	0.0%	0.0%
	**hsa-let-7d**	0.0%	1.0%	0.0%	0.0%	**100.0%**	0.0%	0.0%	0.0%	0.0%
	**hsa-let-7e**	0.0%	0.9%	0.0%	0.0%	0.0%	**100.0%**	0.2%	0.1%	0.0%
	**hsa-let-7f**	3.8%	1.4%	0.0%	0.8%	0.0%	0.0%	**100.0%**	0.1%	0.0%
	**hsa-let-7g**	0.0%	0.1%	0.0%	0.0%	0.5%	0.0%	0.0%	**100.0%**	10.3%
	**hsa-let-7i**	0.0%	0.2%	0.1%	0.0%	0.0%	0.0%	0.0%	0.9%	**100.0%**

Supplementary Table 6 hsa-let-7 miRNA sequence comparison chart. The nucleotide different from hsa-let-7a was marked in red.

	Sequence of mature miRNA
**hsa-miR-98**	ugagguaguaa guuguauuguu
**hsa-let-7a**	ugagguaguag guuguauaguu
**hsa-let-7b**	ugagguaguag guugugugguu
**hsa-let-7c**	ugagguaguag guuguaugguu
**hsa-let-7d**	agagguaguag guugcauaguu
**hsa-let-7e**	ugagguaggag guuguauaguu
**hsa-let-7f**	ugagguaguag auuguauaguu
**hsa-let-7g**	ugagguaguag uuuguacaguu
**hsa-let-7i**	tgaggtagtag tttgtgctgtt

Supplementary Table 7 Oligonucleotides used in miRFLP miR-92ab assay. The sequences of omega primers, targeted miRNA or iRSC molecules, the sequences of 3´ adapters and the expected amplicon sizes are listed below.

RNA Name	Omega Primer	miRNA sequence	RNA Copy per Rx	3' Adapter	Fragment Length
	5' – 3'	5' – 3'		5' – 3'	expected
**iRCS #4**	GTGCTGAGTCACGAGGTATTCTA T GGCACGCTTTCATTAGCGTGCC TCGGATTATGA	ACCGUACAUCU UCAUAAUCCGA	3.75 X 10^5	CACCGACAGGAGACCTGTTCT ACCGTACATCT	91.01nt
**iRCS #5**	GTGCTGAGTCACGAGGTATTCTA TGTTCTT GGCACGCTTTCATTAGCGTGCC TCGGATATGCA	ACCGUACAUCU UGCAUAUCCGA	3.75 X 10^4		96.90nt
**iRCS #6**	GTGCTGAGTCACGAGGTATTCTA TGAACTTGAC GGCACGCTTTCATTAGCGTGCC TCGGATTACTA	ACCGUACAUCU UAGUAAUCCGA	3.75 X 10^3		99.89nt
**miR-92b-3p**	GTGCTGAGTCACGAGGTATTCTA TGTTCTTGAG TTATATTCA GGCACGCTTTCATTAGCGTGCC G GAGGCCGGGA	UAUUGCACUCGUCCCGGCCUCC	variable	CACCGACAGGAGACCTGTTCTATAC TATTGCACTCG	113.19nt
**miR-92a-3p**		UAUUGCACUUGUCCCGGCCUGU	variable	CACCGACAGGAGACCTGTTCTATAC ATC TATTGCACTTG	116.20nt

*: Average MW (308.95 Delton) of dG, dC, dA and dT is counted as 1 nt. MW of 5' Fam: 474.5.

Supplementary Table 8 Oligonucleotides used in miRFLP IM assay. The sequences of omega primers, the targeted miRNA or iRSC molecules, the sequences of 3´ adapters and the expected amplicon sizes are listed below.

RNA Name	Omega Primer 5' – 3'	miRNA sequence 5' – 3'	RNA Copy per RT	3' Adapter 5' – 3'	Fragment Length expected
**iRCS #1**	GTGCTGAGTCACGAGGTATTCTA TGAATACCTTCAACTTGCAGTTACTGCAAG TCAT GGCACGCTTCTTAGCGTGCC TCGGATATGCAA	ACCGUACAUCU ugu UGCAUAUCCGA	3 X 10^6	CACGGAGGTGTTATCCGAAGA ATACTAA ACCGTACATC	129.95nt
**iRCS #2**		ACCGUACAUCU ugaagu UGCAUAUCCGA	5 X 10^5		133.04nt
**iRCS #3**		ACCGUACAUCU ugaaucagu UGCAUAUCCGA	8.3 X 10^4		135.97nt
**RNA spike-in**		ACCGUACAUCU UCAUAAUCCGA	variable		126.91nt
**hsa-miR-181a-5p**	GTGCTGAGTCACGAGGTATTCTAACTTAGGCACGCTT CTTAGCGTGCC ACTCACCGACA	AACAUUCAACGCUGUCGGUGAGU	variable	CACGGAGGTGTTATCCGAAGA AACATTCAACG	91.71nt
**hsa-miR-150-5p**	GTGCTGAGTCACGAGGTATTCTATCTTATGCGGCACG CTTCTTAGCGTGCC CACTGGTACAA	UCUCCCAACCCUUGUACCAGUG	variable	CACGGAGGTGTTATCCGAAGA TCTCCCAACC	94.15nt
**hsa-miR-146a-5p**	GTGCTGAGTCACGAGGTATTCTATGAATACCTTCGGC ACGCTTCTTAGCGTGCC AACCCATGGAA	UGAGAACUGAAUUCCAUGGGUU	variable	CACGGAGGTGTTATCCGAAGA TGAGAACTGAA	96.51nt
**hsa-miR-222-3p**	GTGCTGAGTCACGAGGTATTCTACATTCACCTAGAAT GTAGAGGGCACGCTTCTTAGCGTGCC ACCCAGTAGC	AGCUACAUCUGGCUACUGGGU	variable	CACGGAGGTGTTATCCGAAGA AGCTACATCTG	104.83nt
**hsa-miR-146b-5p**	GTGCTGAGTCACGAGGTATTCTATGTTCTTGCAGTTAA GGTTAGTAGGCACGCTTTCATTAGCGTGCC AGCCTATGGAA	UGAGAACUGAAUUCCAUAGGCU	variable	CACGGAGGTGTTATCCGAAGA TGAGAACTGAA	110.93nt

*: Average MW (308.95 Delton) of dG, dC, dA and dT is counted as 1 nt. MW of 5' Fam: 474.5.

Supplementary Table 9 Incubation duration affects the quantification of serum miRNAs. The incubation time alters miRNA quantification. The incubation duration between 30 s and 1 m at 95ºC yields the best miRNA quantification.

Heat Treatment at 95C		miR-181a	miR-150	miR-146a	miR-222	miR-146b
S42 serum	30 s	182885	40539	688735	96205	809
	1 m	138575	12769	422531	40750	1079
	2 m	73852	15063	212598	8877	328
	3 m	26835	4076	100397	7549	247
	10 m	6762	4210	133	10	8
S43 serum	30 s	56831	9136	93419	13101	171
	1 m	39802	20072	113184	45267	0
	2 m	0	16817	69351	4717	6
	3 m	0	3554	36005	4406	9
	10 m	0	4239	0	0	11

unit: miRNA copy number / µl serum.

Supplementary Table 10 Optimization of serum-direct miRFLP assay. Serum samples were diluted in serum lysis buffer containing 0.25% Tween-20. Serum lysates were incubated at different temperatures and different durations before miRNA measurements.

	miR-181a	miR-150	miR-146a	miR-222	miR-146b	miR-181a	miR-150	miR-146a	miR-222	miR-146b
Serum treated at 75°C for 5 min						Coefficency of variation (%)				
S1 Serum	53621	3564	128148	30692	521	30.9%	8.2%	13.0%	31.6%	51.6%
S2 Serum	17348	2829	40143	13580	53	2.9%	21.6%	18.7%	59.0%	16.2%
S3 Serum	59054	8225	122215	36814	892	16.7%	2.7%	12.6%	37.3%	67.2%
S4 Serum	267339	20876	404813	90553	1330	42.0%	20.8%	3.2%	15.3%	24.4%
Serum treated at 80°C for 3 min						Coefficency of variation (%)				
S1 Serum	140292	4227	209998	53093	313	45.7%	25.2%	18.2%	50.9%	118.6%
S2 Serum	26875	3719	50973	18698	6	23.7%	26.9%	13.3%	15.2%	86.9%
S3 Serum	57106	6931	117133	26990	426	13.2%	18.5%	15.2%	15.8%	45.2%
S4 Serum	291024	36999	466588	119665	1000	41.6%	12.7%	5.2%	2.3%	35.9%
Serum treated at 90°C for 1 min						Coefficency of variation (%)				
S1 Serum	115240	7540	190799	34523	302	34.5%	53.8%	12.2%	3.2%	89.0%
S2 Serum	24792	3599	56045	17479	42	47.2%	19.4%	7.0%	44.4%	135.2%
S3 Serum	178371	18710	268331	48058	887	38.4%	24.3%	23.9%	63.3%	44.2%
S4 Serum	554759	48406	508015	112940	802	35.2%	27.4%	7.5%	35.8%	49.1%

unit: miRNA copy number / µl serum.

Supplementary Table 11 Correlation coefficients of tissue-enriched miRNAs and RBC-associated miRNAs in sera of rats over the time course of CCl4 treatment. (a) control rat #11. (b) rat #8 treated with CCl4.

a

Correlations

		miR-9	miR-122	miR-192a	miR-92a	miR-451
miR-9	Pearson Correlation	.[a]	.[a]	.[a]	.[a]	.[a]
	Sig. (2-tailed)		.	.	.	.
	N	5	5	5	5	5
miR-122	Pearson Correlation	.[a]	1	-.750	.351	.025
	Sig. (2-tailed)	.		.144	.562	.969
	N	5	5	5	5	5
miR-192a	Pearson Correlation	.[a]	-.750	1	.195	.391
	Sig. (2-tailed)	.	.144		.753	.515
	N	5	5	5	5	5
miR-92a	Pearson Correlation	.[a]	.351	.195	1	.911*
	Sig. (2-tailed)	.	.562	.753		.032
	N	5	5	5	5	5
miR-451	Pearson Correlation	.[a]	.025	.391	.911*	1
	Sig. (2-tailed)	.	.969	.515	.032	
	N	5	5	5	5	5

*. Correlation is significant at the 0.05 level (2-tailed).

a. Cannot be computed because at least one of the variables is constant.

b

Correlations

		miR-9	miR-122	miR-192a	miR-92a	miR-451
miR-9	Pearson Correlation	1	.991**	.981**	-.220	-.103
	Sig. (2-tailed)		.001	.003	.723	.869
	N	5	5	5	5	5
miR-122	Pearson Correlation	.991**	1	.997**	-.263	-.171
	Sig. (2-tailed)	.001		.000	.669	.783
	N	5	5	5	5	5
miR-192a	Pearson Correlation	.981**	.997**	1	-.259	-.188
	Sig. (2-tailed)	.003	.000		.673	.762
	N	5	5	5	5	5
miR-92a	Pearson Correlation	-.220	-.263	-.259	1	.959**
	Sig. (2-tailed)	.723	.669	.673		.010
	N	5	5	5	5	5
miR-451	Pearson Correlation	-.103	-.171	-.188	.959**	1
	Sig. (2-tailed)	.869	.783	.762	.010	
	N	5	5	5	5	5

**. Correlation is significant at the 0.01 level (2-tailed).

Supplementary Table 12 The responses of ABI 3730xl DNA analyzer detector. An arbitrary PCR sample with multiple fragments was diluted by 25, 50, 100, 200 and 400 folds in 1xTE and the diluted samples were analyzed in triplicates by ABI 3730xl DNA analyzers. Eleven peaks with fluorescence abundance ranging from 21 FU – 24949 FU were picked for regression analyses. The quadratic regression and power regression models were found as the best fit across all peaks with coefficients >0.98 respectively. FU were read for each dilution.

		**PCR fragment Length**										
	**Dilution Factor**	**52.87 nt**	**62.00 nt**	**88.98 nt**	**91.16 nt**	**92.09 nt**	**97.51 nt**	**103.18 nt**	**108.35 nt**	**111.25 nt**	**124.06 nt**	**126.00 nt**
**Repeat #1**	**25x**	3400	253	13137	21096	12402	688	570	9599	6101	328	322
	**50x**	2180	172	8881	14113	8308	459	337	6253	3966	220	213
	**100x**	1190	89	4852	7807	4683	250	191	3546	2175	123	130
	**200x**	640	53	2594	4197	2512	140	104	1877	1125	59	67
	**400x**	304	23	1211	1997	1180	70	50	918	552	36	
	**R square**	**0.995**	**0.999**	**0.993**	**0.992**	**0.993**	**0.997**	**0.998**	**0.993**	**0.994**	**0.999**	**0.998**
**Repeat #2**	**25x**	3501	265	13147	21005	11907	694	482	9494	5886	317	351
	**50x**	2171	163	8195	13460	7643	444	325	6076	3647	207	210
	**100x**	1235	93	4747	7570	4275	260	186	3328	2019	120	121
	**200x**	582	46	2210	3569	2021	114	86	1552	905	48	60
	**400x**	257	21	1544	1890	942	66	44	1060	571	34	40
	**R square**	**0.994**	**1.000**	**0.987**	**0.988**	**0.990**	**0.997**	**0.998**	**0.988**	**0.991**	**0.998**	**0.998**
**Repeat #3**	**25x**	4015	312	15315	24949	14592	813	725	11584	7171	382	394
	**50x**	2540	196	10055	15981	9542	538	380	7187	4495	247	245
	**100x**	1301	98	5191	8263	4872	285	203	3646	2274	117	136
	**200x**	726	61	2945	4660	2820	166	114	2037	1256	74	78
	**400x**	307	30	1259	2052	1176	70	53	888	563	34	52
	**R square**	**0.993**	**0.999**	**0.989**	**0.987**	**0.989**	**0.996**	**0.998**	**0.990**	**0.991**	**0.999**	**0.996**
